# Supplementary material for: Complex‐centric proteome profiling by SEC‐SWATH‐MS
Source: Mol Syst Biol. 2019 Jan 14;15(1):e8438. doi: 10.15252/msb.20188438 (PMC6346213; doi:10.15252/msb.20188438)
Supplement: Supplementary file 8 — Dataset EV7 [file MSB-15-e8438-s008.zip › feature_plots_string/Q9BVI4.pdf]

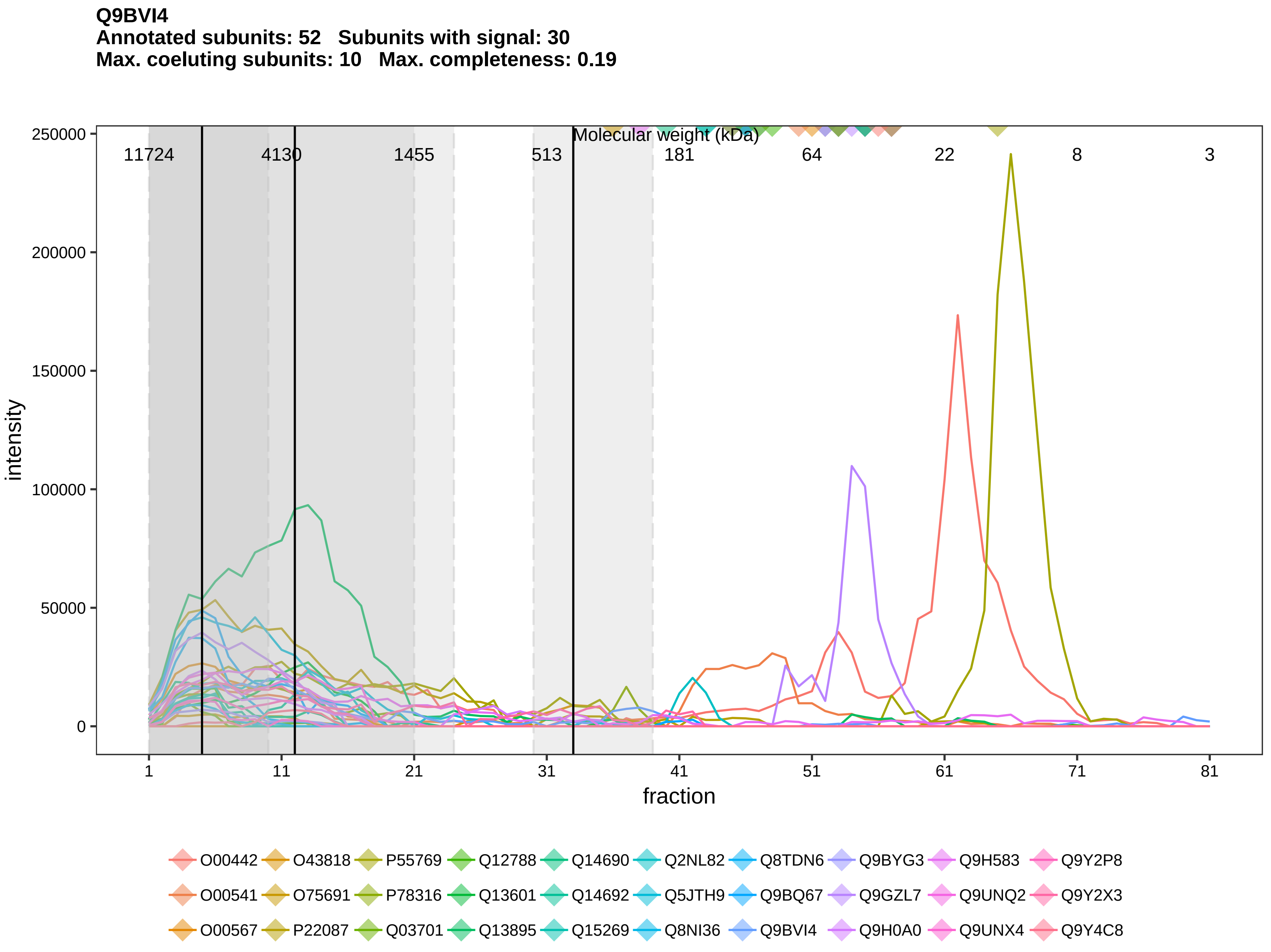
Q9BVI4
Annotated subunits: 52 Subunits with signal: 30
Max. coeluting subunits: 10 Max. completeness: 0.19
Molecular weight (kDa)
11724
4130
1455
513
181
64
22
8
3
intensity
250000
200000
150000
100000
50000
0
1
11
21
31
41
51
61
71
81
fraction
O00442
O43818
P55769
Q12788
Q14690
Q2NL82
Q8TDN6
Q9BYG3
Q9H583
Q9Y2P8
O00541
O75691
P78316
Q13601
Q14692
Q5JTH9
Q9BQ67
Q9GZL7
Q9UNQ2
Q9Y2X3
O00567
P22087
Q03701
Q13895
Q15269
Q8NI36
Q9BVI4
Q9H0A0
Q9UNX4
Q9Y4C8
